# Dietary selenium intake in relation to non-alcoholic fatty liver disease assessed by fatty liver index and hepatic steatosis index; a cross-sectional study on the baseline data of prospective PERSIAN Kavar cohort study.

**DOI:** 10.1186/s12902-023-01307-4

**Published:** 2023-03-03

**Authors:** Sara Shojaei Zarghani, Nima Rahimi Kashkooli, Zahra Bagheri, Mahdy Tabatabaei, Mohammad Reza Fattahi, Ali Reza Safarpour

**Affiliations:** 1 Colorectal Research Center, Shiraz University of Medical Sciences, Shiraz, Iran; 2 Internal Medicine Department, School of Medicine, Shiraz University of Medical Sciences, Shiraz, Iran; 3 Department of Biostatistics, School of Medicine, Shiraz University of Medical Sciences, Shiraz, Iran; 4 School of Medicine, Shiraz University of Medical Sciences, Shiraz, Iran; 5 Gastroenterohepatology Research Center, Shiraz University of Medical Sciences, Shiraz, Iran

**Keywords:** Selenium, Non-alcoholic fatty liver disease, PERSIAN Kavar cohort study

## Abstract

**Background:**

There is limited and conflicting evidence on the association between selenium and non-alcoholic fatty liver disease (NAFLD). Therefore, the present population-based cross-sectional study aimed to explore the relationship between dietary selenium intake and the risk of NAFLD.

**Methods:**

A total of 3026 subjects from the PERSIAN (Prospective Epidemiological Research Studies in IrAN) Kavar cohort study were included in the analysis. The daily selenium intake was evaluated using a semi-quantitative food frequency questionnaire, and energy-adjusted quintiles of selenium intake (µg/day) were calculated. NAFLD was defined as the fatty liver index (FLI) ≥ 60 or the hepatic steatosis index (HSI) > 36. The association between dietary selenium intake and NAFLD was evaluated using logistic regression analysis.

**Results:**

The prevalence rates of NAFLD were 56.4% and 51.9%, based on the FLI and HSI markers, respectively. The odds ratios (ORs) for FLI-defined NAFLD were 1.31 (95% confidence interval (CI): 1.01–1.70) and 1.50 (95% CI: 1.13–1.99) for the fourth and fifth quintiles of selenium intake, respectively, after adjustment for sociodemographic variables, smoking status, alcohol drinking, physical activity, and dietary factors (*P* trend = 0.002). There was also a similar association between selenium intakes and HSI-defined NAFLD (OR = 1.34 (95% CI: 1.03–1.75) for the fourth quintile and OR = 1.50 (95% CI: 1.12–2.01) for the fifth quintile of selenium intake) (*P* trend = 0.006).

**Conclusion:**

In this large sample study, we observed a weak positive association between dietary selenium intake and NAFLD risk.

## Introduction

Non-alcoholic fatty liver disease (NAFLD) encompasses a variety of hepatic disorders, including steatosis, steatohepatitis, fibrosis, cirrhosis, and hepatocellular carcinoma [1]. The global prevalence of NAFLD is 25.24%, with the highest rates related to the Middle East and South America [2]. This disorder is also becoming one of the most common causes of liver transplantation [3]. NAFLD is a hepatic manifestation of metabolic syndrome and is strongly associated with obesity, insulin resistance, type 2 diabetes mellitus, and dyslipidemia [4]. Previous studies suggest that some dietary and lifestyle factors could influence NAFLD pathogenesis, prevention, and treatment [5, 6].

Selenium is a trace mineral and an essential component of the active sites of several proteins, such as glutathione peroxidase, thioredoxin reductase, selenoprotein P, and iodothyronine deiodinase [7, 8]. Therefore, this micronutrient participates in numerous body functions, including cell signaling systems, defending against free radicals, modulation of inflammatory responses, and immune and reproductive systems regulations [7, 9]. There are two forms of selenium in nature and organisms. Selenomethionine and selenocysteine are organic, and selenide, selenite, selenate, and elemental selenium are inorganic forms [8, 10]. The primary source of dietary selenium in humans is selenomethionine [7]. The Recommended Dietary Allowance (RDA) for selenium is 55 µg/ day for adults [11]. The amount of selenium in foods, especially plant-based foods, depends on the selenium content of the soil in a specific geographical area; therefore, dietary selenium intake varies significantly between countries. However, meat and dairy products, eggs, cereals, fish, poultry, seafood, and Brazil nuts are the main sources, and plants are the poor sources of selenium [8].

There are conflicting epidemiological studies on the association between selenium and metabolic disorders. Higher selenium intake and blood levels have been associated with an elevated risk of diabetes [12–16], hyperlipidemia [17, 18], hypertension [19], and NAFLD [20, 21]. Nonetheless, some evidence suggested no or a negative association between selenium and the risk of NAFLD or diabetes [22–24]. Therefore, due to the limited evidence and conflicting data, we aimed to perform the present cross-sectional study to investigate the association between dietary selenium intake and NAFLD in the general population of Kavar County.

## Materials and methods

### Study design and population

The data utilized in the current cross-sectional study was obtained from the baseline phase of the PERSIAN Kavar cohort study (PKCS), a prospective cohort aimed to assess the prevalence, trends, and risk factors of non-communicable diseases with a baseline phase between 2017 and 2019. The PKCS involves 4997 individuals comprising 2419 men and 2578 women aged 35 to 70 living in the urban area of Kavar County, Fars province, Iran [25]. All participants signed informed written consent. The present study was performed in line with the principles of the Declaration of Helsinki and was approved by the Ethics Committee of Shiraz University of medical sciences, Shiraz, Iran (Code: IR.SUMS.REC.1401.142).

**Fig. 1:**
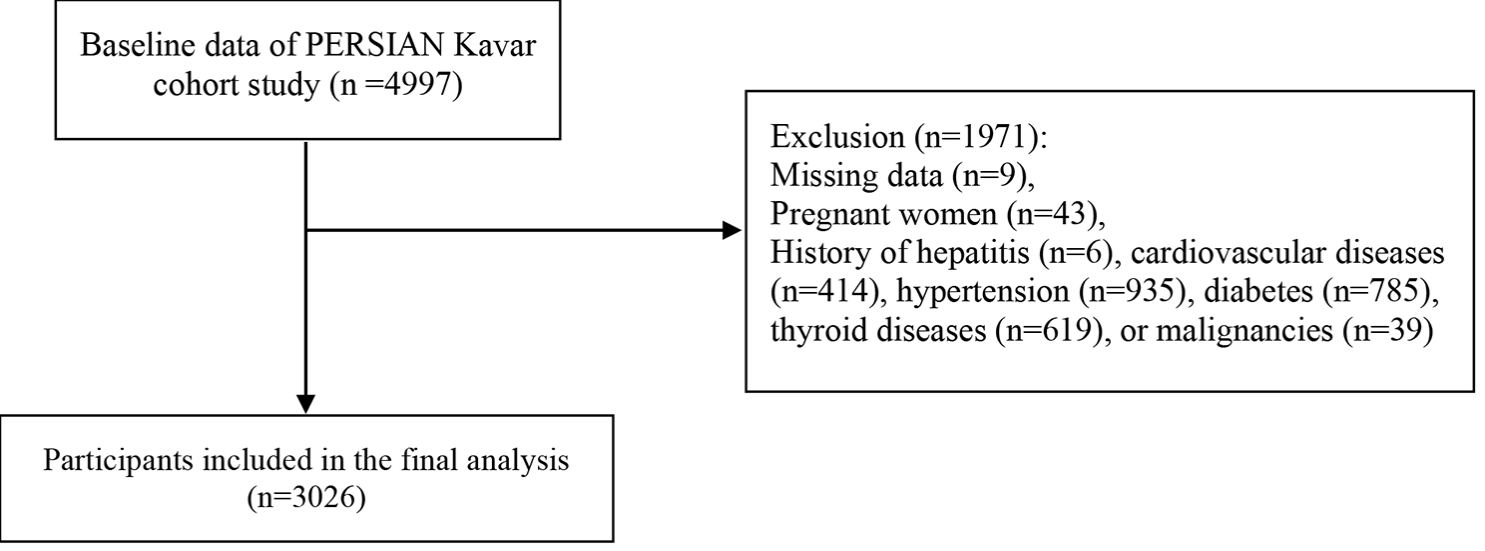
Flow diagram of the study participants

For the current analysis, we first excluded a total of 1971 participants who met the following exclusion criteria: missing data for laboratory tests (n = 9), being pregnant (n = 43), or having a history of hepatitis (n = 6), cardiovascular diseases (n = 414), hypertension (n = 935), diabetes (n = 785), thyroid diseases (n = 619), or malignancies (n = 39). None of the individuals reported implausible total energy intake (< 800 or > 8000 kcal/d for men and < 600 or > 6000 kcal/d for women) [26] or heavy alcohol intake (> 21 drinks per week in men and > 14 drinks per week in women) [27]. Finally, 3026 participants were included in our study (Fig. 1).

### Dietary intake and outcome assessment

Dietary intakes were evaluated using a validated and semi-quantitative food frequency questionnaire (FFQ) with 130 items. Four trained nutritionists conducted the nutritional interview and completed the questionnaire regarding the participant’s usual dietary intakes during the previous year, using a food album and scales. The participants were requested to report the amount and frequency of consumption of each food item on a day, week, month, or year, according to the standard serving sizes. The daily intake of nutrients were calculated by multiplying the frequency of consumption of each food item and the nutrient content of that specific item and then summing amounts across all relevant food items [28]. Selenium intake was adjusted for total energy intake using the residual method [29].

FLI and HSI, two valid markers defined below, were calculated for NAFLD prediction. The FLI ≥ 60 or HSI > 36 was considered NAFLD.

FLI=(e^0·953×log^_e_^(triglycerides (TG))+0·139×body mass index (BMI)+0·718×log^_e_^(gamma−glutamyltransferase(GGT))+0·053×waist circumference–15·745^)/(1 + e^0·953×log^_e_^(TG)+0·139×BMI+0·718×log^_e_^(GGT)+0·053×waist circumference–15·745^)×100 [30].

HSI = 8×(alanine aminotransferase (ALT)/aspartate aminotransferase (AST) ratio) + BMI (+ 2, if female) [31].

### Other variables

Information about sociodemographic features, medical history, physical activity (in the past year), alcohol intake, and smoking was collected through interviews using general and medical questionnaires. Socioeconomic status was assessed by the wealth score index (WSI) based on households’ assets. The anthropometric components (height, weight, and waist circumference) and blood pressure were measured by a physician and trained staff [25, 32]. BMI also was calculated as weight (kg) divided by height (meter) squared. Venous blood samples were obtained after 10–14 h of fasting state. The measures of serum biochemical parameters (lipid profile, liver enzymes, and fasting plasma glucose (FPG)) were conducted using commercial kits (Pars Azmoon, Iran) by the auto-analyzer (model BT3000 Plus, Biotecnica®, Italy).

### Statistical analysis

IBM SPSS (version 26.0) was used for data analysis. The normality of data distribution was assessed by descriptive statistics. Parametric, non-parametric, and qualitative data are expressed as mean ± standard deviation (SD), median (range), or frequency (percentages), respectively. Between-group differences were determined using the independent sample t-test or analysis of variance (ANOVA) test for parametric variables, Mann–Whitney U or Kruskal–Wallis tests for non-parametric parameters, and Chi-square test for categorical variables. Logistic regression analysis was carried out to disclose the independent association between quintiles of energy-adjusted selenium intake and NAFLD risk according to the two adjusted models. A test for linear trend was done by including dietary selenium as a continuous variable in the previous models. Values are expressed as odds ratio (OR) and 95% confidence interval (CI). A two-sided *P*-value < 0.05 was considered significant.

## Results

Table 1 shows the characteristics of the study participants by quintiles of dietary energy-adjusted selenium intake. The median age of the total study population was 45 years (minimum: 35, maximum: 70). Subjects in the highest quintile of energy-adjusted selenium intake were more likely to be male, Turk Nomad, to consume lower fiber, fructose, and saturated fatty acids, and to have lower age and higher physical activity, blood pressure, TG, ALT, AST, and GGT levels compared to those in the lowest quintile. Furthermore, participants with higher selenium intake had lower high-density and low-density lipoprotein cholesterol (HDL-C and LDL-C). The prevalence of NAFLD in the total population was 56.4% according to the FLI and 51.9% based on the HSI markers. The NAFLD prevalence was not significantly different between quintiles of selenium intake.

**Table 1:** Basic characteristics of the participants according to the quintiles of energy-adjusted selenium intake

Basic characteristics	Quintiles of energy-adjusted selenium intake						*P*-value
	All	Q1	Q2	Q3	Q4	Q5	
n	3026	605	605	605	606	605	-
Sex, n (%)							
Male Female	1716 (56.7) 1310 (43.3)	317 (52.4) 288 (47.6)	296 (48.9) 309 (51.1)	306 (50.6) 299 (49.4)	360 (59.4) 246 (40.6)	437 (72.2) 168 (27.8)	< 0.001
Education, n (%)							
Illiterate Elementary school Middle and high school College	737 (24.4) 929 (30.7) 1043 (34.5) 317 (10.5)	155 (25.6) 207 (34.2) 201 (33.2) 42 (6.9)	156 (25.8) 193 (31.9) 194 (32.1) 62 (10.2)	128 (21.2) 198 (32.7) 197 (32.6) 82 (13.6)	171 (28.2) 164 (27.1) 211 (34.8) 60 (9.9)	127 (21.0) 167 (27.6) 240 (39.7) 71 (11.7)	< 0.001
Ethnicity, n (%)							
Persian Turk Nomad Others or mixed	2290 (75.7) 621 (20.5) 115 (3.8)	478 (79.0) 100 (16.5) 27 (4.5)	470 (77.7) 109 (18.0) 26 (4.3)	482 (79.7) 104 (17.2) 19 (3.1)	441 (72.8) 136 (22.4) 29 (4.8)	419 (69.3) 172 (28.4) 14 (2.3)	< 0.001
Smoking, n (%)							
Non-smoker Ex-smoker Current smoker	2212 (73.1) 220 (7.3) 594 (19.6)	427 (70.6) 46 (7.6) 132 (21.8)	467 (77.2) 27 (4.5) 111 (18.3)	468 (77.4) 42 (6.9) 95 (15.7)	440 (72.6) 46 (7.6) 120 (19.8)	410 (67.8) 59 (9.8) 136 (22.5)	< 0.001
Alcohol intake, n (%)							
No Yes	2684 (88.7) 342 (11.3)	531 (87.8) 74 (12.2)	550 (90.9) 55 (9.1)	558 (92.2) 47 (7.8)	534 (88.1) 72 (11.9)	511 (84.5) 94 (15.5)	< 0.001
Wealth score index, n (%)							
1st quintile 2nd quintile 3rd quintile 4th quintile 5th quintile	614 (20.3) 618 (20.4) 620 (20.5) 661 (21.8) 513 (17.0)	106 (17.5) 120 (19.8) 115 (19.0) 153 (25.3) 111 (18.3)	111 (18.3) 124 (20.5) 135 (22.3) 119 (19.7) 116 (19.2)	106 (17.5) 114 (18.8) 117 (19.3) 142 (23.5) 126 (20.8)	125 (20.6) 128 (21.1) 135 (22.3) 129 (21.3) 89 (14.7)	166 (27.4) 132 (21.8) 118 (19.5) 118 (19.5) 71 (11.7)	< 0.001
Age (years), mean ± SD	46.00 ± 8.17	47.08 ± 8.24	46.23 ± 8.10	45.89 ± 8.15	46.15 ± 8.46	44.66 ± 7.72	< 0.001
BMI (kg/m^2^), mean ± SD	26.67 ± 4.68	26.73 ± 4.96	26.55 ± 4.98	26.76 ± 4.46	26.67 ± 4.49	26.64 ± 4.50	0.947
Waist circumference (cm), mean ± SD	93.92 ± 10.60	94.29 ± 11.21	93.88 ± 10.98	94.14 ± 10.43	93.76 ± 10.17	93.53 ± 10.22	0.744
Serum TC (mg/dl), mean ± SD	174.81 ± 36.00	176.68 ± 34.27	173.40 ± 36.00	176.79 ± 35.41	175.07 ± 35.98	172.08 ± 38.08	0.095
Serum HDL-C (mg/dl), mean ± SD	41.73 ± 9.17	42.33 ± 9.33	43.04 ± 9.23	41.72 ± 9.43	41.49 ± 9.11	40.07 ± 8.49	< 0.001
Serum LDL-C (mg/dl), mean ± SD	103.77 ± 29.56	106.34 ± 27.95	102.53 ± 30.07	106.06 ± 28.69	104.26 ± 29.80	99.64 ± 30.78	< 0.001
Serum TG (mg/dl), median (range)	123.00 (1408)	119.00 (652)	126.00 (900)	126.00 (889)	126.00 (709)	133.00 (1408)	0.004
FPG (mg/dl), median (range)	93.00 (281)	93.00 (279)	92.00 (146)	93.00 (214)	93.00 (127)	93.00 (272)	0.863
ALT (U/L), median (range)	17.00 (218)	16.00 (95)	16.00 (180)	17.00 (204)	18.00 (157)	19.00 (215)	< 0.001
AST (U/L), median (range)	17.00 (396)	17.00 (57)	16.00 (395)	17.00 (77)	17.00 (122)	18.00 (174)	< 0.001
GGT (U/L), median (range)	19.00 (370)	19.00 (153)	18.00 (143)	18.00 (257)	20.00 (161)	21.00 (367)	< 0.001
SBP (mmHg), mean ± SD	114.69 ± 13.24	114.00 ± 13.19	113.64 ± 13.77	114.60 ± 13.51	115.24 ± 13.11	115.98 ± 12.51	0.015
DBP (mmHg), mean ± SD	75.48 ± 9.45	74.51 ± 9.42	74.78 ± 9.73	75.45 ± 9.46	75.79 ± 9.38	76.87 ± 9.11	< 0.001
Activity level (MET-h/week), mean ± SD	41.94 ± 6.91	41.67 ± 6.70	41.72 ± 6.33	41.92 ± 6.61	41.51 ± 6.59	42.86 ± 8.12	0.006
Dietary total energy intake (kcal/d), mean ± SD	2247.02 ± 607.57	2414.00 ± 673.77	2134.43 ± 549.51	2127.85 ± 559.15	2154.81 ± 572.45	2404.19 ± 603.20	< 0.001
Dietary SFA intake (g/day), mean ± SD	19.03 ± 7.66	21.78 ± 9.21	18.84 ± 7.34	17.84 ± 6.78	17.82 ± 6.77	18.86 ± 7.25	< 0.001
Dietary cholesterol intake (mg/day), mean ± SD	234.30 ± 112.60	218.59 ± 97.56	213.19 ± 94.16	255.97 ± 104.93	236.49 ± 109.53	277.27 ± 139.53	< 0.001
Dietary fiber intake (g/day), mean ± SD	26.85 ± 9.58	30.12 ± 11.56	26.02 ± 9.04	25.58 ± 9.00	24.98 ± 8.39	27.56 ± 8.71	< 0.001
Dietary fructose intake (g/day), mean ± SD	30.34 ± 16.90	41.30 ± 22.15	31.34 ± 14.57	28.43 ± 14.23	25.66 ± 13.50	24.99 ± 12.94	< 0.001
Dietary selenium intake (µg/day), mean ± SD	109.29 ± 37.03	88.77 ± 30.49	93.73 ± 27.36	102.91 ± 27.91	114.48 ± 28.79	146.58 ± 38.13	< 0.001
Dietary selenium intake (µg/kg/day), mean ± SD	1.57 ± 0.55	1.29 ± 0.47	1.37 ± 0.42	1.49 ± 0.43	1.64 ± 0.46	2.04 ± 0.61	< 0.001
NAFLD based on FLI, n (%)	1708 (56.4)	336 (55.5)	323 (53.4)	347 (57.4)	350 (57.8)	352 (58.2)	0.424
NAFLD based on HSI, n (%)	1570 (51.9)	309 (51.1)	297 (49.1)	318 (52.6)	323 (53.3)	323 (53.4)	0.526

Abbreviations: ALT: Alanine transaminase, AST: Aspartate transaminase, BMI: Body mass index, DBP: Diastolic blood pressure, FLI: Fatty liver index, FPG: Fasting plasma glucose, GGT: Gamma-Glutamyl-Transferase, HDL-C: High-density lipoprotein cholesterol, HSI: Hepatic steatosis index, LDL-C: Low-density lipoprotein cholesterol, NAFLD: non-alcoholic fatty liver disease, SBP: Systolic blood pressure, SFA: Saturated fatty acids, TC: total cholesterol, TG: triglyceride. Parametric, non-parametric, and categorical data are expressed as mean ± standard deviation (SD), median (range), or frequency (percentages), respectively.

Between-group differences in variables were assessed using the analysis of variance (ANOVA) test for parametric variables, the Kruskal–Wallis test for non-parametric parameters, and the Chi-square test for categorical variables.

The characteristics of the participants according to the NAFLD status are reported in Table 2. The NAFLD patients were more likely to be Persian, female, non-smoker, non-drinker, and have higher socioeconomic status than the healthy subjects. They also had higher BMI, waist circumference, blood pressure, TG, total cholesterol, LDL-C, FPG, ALT, AST, and GGT levels. Significant differences also were observed between patients with and without NAFLD regarding physical activity, HDL-C levels, and fiber and fructose intakes. These results were similar between FLI- and HSI-defined NAFLD, except that patients with HSI-defined NAFLD were significantly younger than healthy subjects (45.11 ± 7.45 vs. 46.97 ± 8.78, *P* < 0.001).

**Table 2:** Basic characteristics of the NAFLD and non-NAFLD population

Basic characteristics	NAFLD status					
	With NAFLD based on FLI	Without NAFLD based on FLI	*P*-value	With NAFLD based on HSI	Without NAFLD based on HSI	*P*-value
n (%)	1708 (56.4)	1318 (43.6)	-	1570 (51.9)	1456 (48.1)	-
Sex, n (%)						
Male Female	873 (51.1) 835 (48.9)	843 (64.0) 475 (36.0)	< 0.001	715 (45.5) 855 (54.5)	1001 (68.8) 455 (31.3)	< 0.001
Education, n (%)						
Illiterate Elementary school Middle and high school Academic degree	415 (24.3) 531 (31.1) 570 (33.4) 192 (11.2)	322 (24.14) 398 (30.2) 473 (35.9) 125 (9.5)	0.289	371 (23.6) 484 (30.8) 545 (34.7) 170 (10.8)	366 (25.1) 445 (30.6) 498 (34.2) 147 (10.1)	0.761
Ethnicity, n (%)						
Persian Turk Nomad Others or mixed	1325 (77.6) 317 (18.6) 66 (3.9)	965 (73.2) 304 (23.1) 49 (3.7)	0.01	1226 (78.1) 283 (18.0) 61 (3.9)	1064 (73.1) 338 (23.2) 54 (3.7)	0.002
Smoking, n (%)						
Non-smoker Ex-smoker Current smoker	1338 (78.3) 117 (6.9) 253 (14.8)	874 (66.3) 103 (7.8) 341 (25.9)	< 0.001	1292 (82.3) 96 (6.1) 182 (11.16)	920 (63.2) 124 (8.5) 412 (28.3)	< 0.001
Alcohol intake						
No Yes	1542 (90.3) 166 (9.7)	1142 (86.6) 176 (13.4)	0.002	1437 (91.5) 133 (8.5)	1247 (85.6) 209 (14.4)	< 0.001
Wealth score index, n (%)						
1st quintile 2nd quintile 3rd quintile 4th quintile 5th quintile	305 (17.9) 322 (18.9) 355 (20.8) 398 (23.3) 328 (19.2)	309 (23.4) 296 (22.5) 265 (20.1) 263 (20.0) 185 (14.0)	< 0.001	299 (19.0) 279 (17.8) 334 (21.3) 358 (22.8) 300 (19.1)	315 (21.6) 339 (23.3) 286 (19.6) 303 (20.8) 213 (14.6)	< 0.001
Age (years), mean ± SD	45.93 ± 7.87	46.10 ± 8.54	0.573	45.11 ± 7.45	46.97 ± 8.78	< 0.001
BMI (kg/m^2^), mean ± SD	29.51 ± 3.74	22.98 ± 2.85	< 0.001	29.87 ± 3.69	23.21 ± 2.81	< 0.001
Waist circumference (cm), mean ± SD	100.22 ± 8.43	85.76 ± 6.94	< 0.001	100.52 ± 8.75	86.81 ± 7.33	< 0.001
Serum TC (mg/dl), mean ± SD	184.12 ± 35.98	162.73 ± 32.25	< 0.001	181.82 ± 35.39	167.24 ± 35.10	< 0.001
Serum HDL-C (mg/dl), mean ± SD	40.38 ± 9.02	43.48 ± 9.07	< 0.001	41.18 ± 9.13	42.32 ± 9.18	0.001
Serum LDL-C (mg/dl), mean ± SD	107.71 ± 30.93	98.66 ± 26.85	< 0.001	107.59 ± 30.28	99.65 ± 28.19	< 0.001
Serum TG (mg/dl), median (range)	154.50 (1381)	93.00 (318)	< 0.001	140.00 (1387)	106.00 (827)	< 0.001
FPG (mg/dl), median (range)	94.00 (280)	91.00 (79)	< 0.001	94.00 (273)	91.00 (281)	< 0.001
ALT (U/L), median (range)	20.00 (218)	14.00 (132)	< 0.001	21.00 (216)	14.00 (182)	< 0.001
AST (U/L), median (range)	17.00 (396)	16.00 (121)	< 0.001	17.00 (174)	16.00 (395)	< 0.001
GGT (U/L), median (range)	23.00 (367)	16.00 (154)	< 0.001	22.00 (369)	17.00 (160)	< 0.001
SBP (mmHg), mean ± SD	117.23 ± 13.18	111.40 ± 12.59	< 0.001	116.39 ± 12.50	112.86 ± 13.77	< 0.001
DBP (mmHg), mean ± SD	77.80 ± 9.07	72.47 ± 9.09	< 0.001	77.34 ± 8.90	73.47 ± 9.62	< 0.001
Activity level (MET-h/week), mean ± SD	41.38 ± 6.38	42.66 ± 7.49	< 0.001	41.39 ± 6.14	42.52 ± 7.62	< 0.001
Dietary total energy intake (kcal/d), mean ± SD	2252.09 ± 606.00	2240.46 ± 609.78	0.602	2242.42 ± 613.61	2251.99 ± 601.16	0.665
Dietary SFA intake (g/day), mean ± SD	18.85 ± 7.38	19.26 ± 8.00	0.146	18.87 ± 7.50	19.20 ± 7.82	0.225
Dietary cholesterol intake (mg/day), mean ± SD	232.62 ± 109.78	236.48 ± 116.15	0.350	231.94 ± 110.09	236.85 ± 115.23	0.231
Dietary fiber intake (g/day), mean ± SD	27.39 ± 9.68	26.15 ± 9.40	< 0.001	27.38 ± 9.89	26.28 ± 9.20	0.002
Dietary fructose intake (g/day), mean ± SD	31.17 ± 16.94	29.28 ± 16.80	0.002	31.01 ± 17.29	29.62 ± 16.45	0.024
Dietary selenium intake (µg/day), mean ± SD	110.03 ± 37.54	108.35 ± 36.34	0.215	109.43 ± 37.76	109.15 ± 36.23	0.838

Abbreviations: ALT: Alanine transaminase, AST: Aspartate transaminase, BMI: Body mass index, DBP: Diastolic blood pressure, FLI: Fatty liver index, FPG: Fasting plasma glucose, GGT: Gamma-Glutamyl-Transferase, HDL-C: High-density lipoprotein cholesterol, HSI: Hepatic steatosis index, LDL-C: Low-density lipoprotein cholesterol, NAFLD: non-alcoholic fatty liver disease, SBP: Systolic blood pressure, SFA: Saturated fatty acids, TC: total cholesterol, TG: triglyceride. Parametric, non-parametric, and categorical data are expressed as mean ± standard deviation (SD), median (range), or frequency (percentages), respectively.

Between-group differences in variables were determined using an independent sample t-test for parametric variables, Mann–Whitney U test for non-parametric parameters, and the Chi-square test for categorical variables.

The association between dietary energy-adjusted selenium intake and NAFLD risk is demonstrated in Table 3. As shown in model 1 (crude ORs), the relationship between selenium intake and NAFLD risk was non-significant in both NAFLD prediction models. After adjustment for age, sex, ethnicity, education levels, smoking status, alcohol intake, WSI, and physical activity (model 2), the adjusted OR and 95% CIs for FLI- and HSI- defined NAFLD comparing the fifth quintile of selenium intake with reference group were 1.42 (1.11–1.80) and 1.40 (1.09–1.79), respectively, with a progressive increase in risk across quintiles (*P* trend < 0.05).

**Table 3:** Risk of NAFLD according to the quintiles of energy-adjusted selenium intake (µg/day)

Models	Q1	Q2	Q3	Q4	Q5	P-trend
NAFLD assessed by FLI						
Event/Total	336/605	323/605	347/605	350/606	352/605	
^†^Model 1, OR (95%CI)	1.00 (Ref.)	0.92 (0.73–1.15)	1.08 (0.86–1.35)	1.09 (0.87–1.37)	1.11 (0.89–1.40)	0.160
^‡^Model 2, OR (95%CI)	1.00 (Ref.)	0.90 (0.71–1.13)	1.05 (0.83–1.32)	1.18 (0.93–1.49)	1.42 (1.11–1.80)	0.001
^§^Model 3, OR (95%CI)	1.00 (Ref.)	0.98 (0.77–1.25)	1.15 (0.90–1.48)	1.31 (1.01–1.70)	1.50 (1.13–1.99)	0.002
**NAFLD assessed by HSI**						
Event/Total	309/605	297/605	318/605	323/606	323/605	
^†^Model 1, OR (95%CI)	1.00 (Ref.)	0.92 (0.74–1.16)	1.06 (0.85–1.33)	1.09 (0.87–1.37)	1.10 (0.88–1.37)	0.336
^‡^Model 2, OR (95%CI)	1.00 (Ref.)	0.86 (0.67–1.09)	0.98 (0.77–1.25)	1.18 (0.93–1.50)	1.40 (1.09–1.79)	0.003
^§^Model 3, OR (95%CI)	1.00 (Ref.)	0.95 (0.74–1.22)	1.11 (0.86–1.43)	1.34 (1.03–1.75)	1.50 (1.12–2.01)	0.006

Abbreviations: CI: confidence interval, BMI: body mass index, FLI: Fatty liver index, HSI: Hepatic steatosis index, NAFLD: non-alcoholic fatty liver disease, OR: odds ratio, Ref.: referent values

Adjusted ORs and 95% CI were determined by multivariable logistic regression

†Model 1: crude and unadjusted; ‡Model 2: adjusted for age, sex, ethnicity, education levels, smoking status, alcohol intake (yes, no), wealth score index, and physical activity; §Model 3: further adjusted for energy, saturated fatty acids, cholesterol, fiber, and fructose intakes

After adjusting for energy, saturated fatty acids, cholesterol, fiber, and fructose intakes (model 3), the multivariable-adjusted ORs and 95% CIs for FLI-defined NAFLD were 0.98 (0.77–1.25), 1.15 (0.90–1.48), 1.31 (1.01–1.70), and 1.50 (1.13–1.99) from the second to the fifth dietary selenium quintile, respectively, compared to the lowest category (*P* trend = 0.002). Furthermore, the multivariable-adjusted ORs and 95% CIs for HSI-defined NAFLD from the second to the fifth quintile were 0.95 (0.74–1.22), 1.11 (0.86–1.43), 1.34 (1.03–1.75), and 1.50 (1.12–2.01), respectively (*P* trend = 0.006) (Table 3).

## Discussion

We conducted a population-based cross-sectional study in a large sample of Kavar County with the primary objective of assessing the relationship between dietary selenium intake and NAFLD prevalence. Our results demonstrated a weak positive linear association between dietary selenium intake and NAFLD risk. The prevalence rates of NAFLD in our population were 56.4% and 51.9%, based on the FLI and HSI markers, respectively. In our study, the mean selenium intake (109.29 µg/day) was higher than the RDA level as well as its intake by other Iranian populations [33]. Moreover, all subjects consumed below the tolerance limits (400 µg/day), and only 3.4% ingested less than the RDA level for selenium intake.

In our study, participants with the highest selenium intake had 50% higher NAFLD risk after adjustment for major confounders. Limited epidemiological investigations have explored the association between selenium and NAFLD prevalence. Liu et al. detected a positive association between more than 121.90 µg/d selenium intake and the odds of steatosis [34]. In another study of 8550 Chinese adults, participants in the third and fourth quartiles of plasma selenium levels had a 72% and 54% increased NAFLD risk compared with those in the reference quartile [20]. Inconsistent with our findings, Wu et al. revealed a positive dose-response relationship between dietary selenium intake, below the recommendations, and the prevalence of NAFLD, detected by ultrasonography, in the general population of China [21]. In another cross-sectional study of 42 adults with NAFLD, a negative and null correlation between selenium intake and liver fat was observed in females and males, respectively [23]. This conflicting evidence could be due to the differences in the population, method of exposure assessment, amounts of selenium intake, method of NAFLD diagnosis, and considered confounding variables.

Many observational investigations demonstrated a positive association between dyslipidemia and diabetes risk with different selenium levels [14, 15, 35, 36]. Insulin resistance and dyslipidemia have fundamental roles in NAFLD development and progression [37]. Therefore, high dietary selenium intake may increase NAFLD risk by dysregulating insulin biosynthesis and secretion and stimulating glucagon secretion, insulin resistance, and dyslipidemia [38]. The increment of liver protein tyrosine phosphatase 1B activity, an enzyme antagonizing insulin signaling and stimulating fatty acid synthesis, is also reported following selenium supplementation [39]. Furthermore, the high intake of this trace element increased hepatic TG by upregulating gluconeogenesis and lipogenesis and downregulating lipolysis in pigs [40]. However, more studies are warranted to clarify other related mechanisms.

Early detection of NAFLD may be helpful for the recognition of those with probably silent progressive NAFLD. Diagnostic routes are different and include clinical, biochemical, and radiographic tests. The liver biopsy remains the gold standard for NAFLD confirmation, but it is practically infeasible as a diagnostic instrument [41]. In our study, NAFLD was predicted by computing FLI and HSI biomarkers. These validated indicators can be used for detecting participants to be referred for lifestyle counseling, ultrasonography, and conducting epidemiologic studies [30, 31]. According to the study by Hsu et al., FLI was a stronger predictor than sex, liver function tests, BMI, body fat, FPG, uric acid, and triglyceride for NAFLD diagnosis in lean patients [42]. In a previous study, a good agreement between NAFLD prevalence by FLI (47.6%) and HSI (53.5%) vs. controlled attenuation parameter derived via transient elastography (CAP-TE) (48.1%) was detected [43]. However, a higher NAFLD prevalence was reported in studies using FLI than ultrasound in obese and diabetic patients [44]. The NAFLD prevalence rates in our studied population were 56.4% and 51.9%, based on the FLI and HSI markers, respectively. These estimations were higher than the prevalence of ultrasonography or liver biopsy-diagnosed NAFLD in Iran (33.95%), yielded by a meta-analysis study published in 2016 [45]. Therefore, differences in NAFLD diagnosis methods and increased incidence of NAFLD in recent years could partly describe the high prevalence of NAFLD in the present study.

The population-based sampling, large sample size, and assessing the association between selenium and NAFLD in the Iranian population for the first time are some strengths of the present study. Nonetheless, there are several limitations. First, due to the specific characteristics of the cross-sectional studies, supposing a causal connection between dietary selenium intake and NAFLD prevalence is impossible. Second, we used FLI and HSI markers but not liver biopsy as the gold standard of NAFLD diagnosis. Third, we did not assess the blood selenium concentration, which provides more reliable evidence regarding selenium’ status in the body. Using FFQ for selenium intake estimation in the current study may cause recall bias and errors in exposure assessment. Fourth, because of the social stigma associated with alcohol consumption in Iranian society, the actual amount of alcohol intake may be biased. Further well-designed prospective cohort studies on the association between blood serum biomarkers and NAFLD risk should be carried out to clarify this association.

## Conclusion

In this study, dietary selenium intake was associated with the prevalence of NAFLD after controlling for major confounders.

## Data Availability

Data supporting the results of this study is available from the author [A.R.S.] upon reasonable request.

## List of abbreviations

ALT: alanine aminotransferase
ANOVA: analysis of variance
AST: aspartate aminotransferase
BMI: body mass index
CI: confidence interval
FFQ: food frequency questionnaire
FLI: fatty liver index
FPG: fasting plasma glucose
GGT: gamma-glutamyltransferase
HDL-C: high-density lipoprotein cholesterol
HSI: hepatic steatosis index
LDL-C: low-density lipoprotein cholesterol
NAFLD: non-alcoholic fatty liver disease
OR: odds ratio
PERSIAN: prospective epidemiological research studies in IrAN
RDA: recommended dietary allowance
SD: standard deviation
TG: triglycerides
WSI: wealth score index
